# Medicinal Plants and Herbal Products From Brazil: How Can We Improve Quality?

**DOI:** 10.3389/fphar.2020.606623

**Published:** 2021-01-27

**Authors:** Rafael M. Palhares, Leopoldo C. Baratto, Marina Scopel, Fernanda. L. B. Mügge, Maria G. L. Brandão

**Affiliations:** ^1^ Centro Especializado em Plantas Aromáticas, Medicinais e Tóxicas (CEPLAMT), Museu de História Natural e Jardim Botânico, Universidade Federal de Minas Gerais, Belo Horizonte, Brazil; ^2^ Laboratório de Farmacognosia Aplicada, Faculdade de Farmácia, Universidade Federal do Rio de Janeiro, Rio de Janeiro, Brazil; ^3^ Laboratório de Farmacognosia, Faculdade de Farmácia, Universidade Federal de Minas Gerais, Belo Horizonte, Brazil; ^4^ Zentrum für Molekulare Biologie, Universität Heidelberg, Heidelberg, Germany

**Keywords:** Brazil, conservation, metabarcoding, resilient plants, traditional knowledge

## Introduction

Brazil has widely diverse flora, rich in medicinal plants, which are an important part of the Amerindian traditional knowledge (Levis et al., 2017). Some Brazilian plants were included decades ago in different Pharmacopoeias because they provide important substances used in medical practice worldwide. Examples are *Carapichea ipecacuanha* (Brot.) L. Andersson (ipecac), source of the emetic and amoebicide alkaloid emetine, and *Pilocarpus microphyllus* Stapf ex Wardlew, source of the antiglaucoma pilocarpine (Nogueira et al., 2010). More recently, Açaí (*Euterpe oleracea* Mart.), native from the Amazon rainforest, became notorious in the international market as a nutraceutical (Carey et al., 2017). Despite its potential, the native vegetation of Brazil has been undergoing intense destruction: all the ecosystems, including the Amazon rainforest, have been quickly replaced by monocultures of sugarcane, soybeans, eucalyptus, and livestock, leading to an intense process of genetic and cultural erosion. On the other side, more recently, the development of bioproducts from Brazilian plants has been stimulated, aiming at a market based in the bioeconomy, which not only brings health benefits but also is important for conservation of biodiversity and consequent mitigation of climate changes (Dinerstein et al., 2020). However, due to the current precarious situation of the herbal products market in Brazil, many steps need to be taken until such a goal is achieved.

## Quality of Herbal Products From Brazil

The denomination herbal products (HP), in Brazil, includes dried plants, sold as tea, and also the finished products used as medicine, nutraceutical, or cosmetics. In this internal market, it is possible to find HP from plant species from three different origins: i) native species that are collected in the local ecosystems, ii) cultivated exotic species, and iii) imported species (mainly dry plants and extracts). HP from i) are mainly commercialized in local markets while HP from ii) and iii) can be found in pharmacies and natural products shops. Since the 1960s, there are regulatory rules for the correct identification of medicinal plants for the commerce in Brazil (Brasil 1967), and more recently, new legislation was launched for regulating the commerce of plants used in traditional knowledge (Carvalho et al., 2018). Despite such efforts, studies done using classical analytical procedures, provided by monographs from Brazilian Pharmacopoeia and Pharmacopoeia from other countries, have shown the existence of serious problems.

Studies with species from the group i) uncovered different problems. Contamination with potentially pathogenic bacteria (enterobacteria and other Gram-negative bacteria) and fungi (*Aspergillus* spp. and *Penicillium* spp.) was found in samples of Brazilian ginseng (*Pfaffia glomerata* (Spreng.) Pedersen and *P. paniculata* Mart.) (Zaroni et al., 2004) and “mate” (*Ilex paraguariensis* A.St.-Hil.) (Borges et al., 2002). Adulteration in leaves of "guaco" (*Mikania glomerata* Spreng. and *M. laevigata* Sch. Bip. ex Bak) was detected in different studies that have shown species substitution and presence of other parts of the plant, insects, and sand (Alvarenga et al., 2009; Melo and Sawaya 2015; Palhares et al., 2015). Samples of “guaraná” (*Paullinia cupana* Kunth) presented low levels of methylxanthines and presence of inorganic materials (sand and earth) and nonessential metals (Araújo et al., 2006; Bara et al., 2006; Sousa et al., 2011). Parts of insects and nonessential minerals were also found in samples of “carqueja” (*Baccharis crispa* Spreng) (Ferrante et al., 2007), “pata-de-vaca” (*Bauhinia forficata* Link) (Engel et al., 2008), and “espinheira-santa” (*Maytenus ilicifolia* Mart. ex Reissek, syn. *Monteverdia ilicifolia* (Mart. ex Reissek) Biral) (Leal et al., 2013). Our group also showed that, from a total of 252 samples of native plants with historical use in Brazilian traditional medicine, purchased from popular markets in all the regions of Brazil, only 50.2% corresponded to the original species (Brandão et al., 2013). It is important to note that *P. glomerata*, *P. paniculata*, *I. paraguariensis*, *M. glomerata*, *M. laevigata*, *P. cupana*, *B. crispa*, *B. forficata*, and *M. ilicifolia* are also commercialized in other countries, suggesting that the problems found in the Brazilian market may be present in the international market as well.

More recently, we added DNA barcoding techniques to the classical analytical studies. One of our studies showed that samples of barks of the Brazilian quina (*Remijia ferruginea* (A.St.-Hil.) DC. and *Strychnos pseudoquina* A. St.-Hil.) were substituted by other species without any correlation to traditional medicine (Palhares et al., 2014). In a similar study, we have analyzed 257 commercial samples of the native species i) *M. ilicifolia* and *Mikania* spp. as well as exotic species ii) *Matricaria recutita* L. and *Passiflora incarnata* L. and imported species iii) *Hamamelis virginiana* L., *Panax ginseng* C. A. Mey, *Peumus boldus* Molina, and *Valeriana officinalis* L. This study showed that substitutions may be as high as 71% (Palhares et al., 2015). In a recent review, Ichim (2019) also showed the results of studies using DNA-based methods for species identification in herbal products commercialized in 37 countries. In Brazil, besides our results (Palhares et al., 2015), the author showed that a study using DNA barcode and a wider range of markers (ITS, trnL, trnL-trnF, psbA-trnH, matK, and rbcL) evidenced substitutions in “quebra-pedra” (*Phyllanthus* spp.) (Inglis et al., 2018). In another study done also with *M. ilicifolia* using PCR-RFLP technique, substitution was also an issue (Nakamura et al., 2013). On the other hand, when analyzing the complete ITS/5.8S region in commercial samples of “Brazilian arnica*”* (*Egletes* *viscosa* (L.) Less), no substitutions were found (Batista et al., 2012). In his review, Ichim concludes that the highest percentage of adulterated commercial HPs among all countries was reported for Brazil.

In another review, Ichim et al. (2020) show results of studies on authenticity using microscopic analysis on 508 herbal medicines and food supplements traded in thirteen countries or territories. All or at least most (>70%) herbal products were reported to be authentic in Argentina, China, Germany, Thailand, and Egypt. In the United States and Peru, a substantial part (>30%) was wrongly declared, and a third group of countries, comprising Iran, Brazil, India, Turkey, and Greece, showed authenticity score lower than 40%. Overall, almost half (49%) of the total products (*n* = 167) microscopically authenticated in Asia were reported to be adulterated, followed by South America (40%) and Europe (39%) and more distantly by North America (33%). Other studies also show that very known and used medicinal plants marketed in Europe, Asia, and the United States such as *Hypericum perforatum* L. (Raclariu et al., 2017), *Echinacea* spp. (Raclariu et al., 2018), and *M. recutita* (Guzelmeric et al., 2017) are often adulterated or show low quality. Other problems were described for Ayurvedic herbal products sold in Norway, Romania, and Sweden (Seethapathy et al., 2019), HP sold in Canada and United States (Newmaster et al., 2013), and Traditional Chinese Medicines (TCM) entering the Australian market (Coghlan et al., 2012), among other countries.

## Discussion

Considering the current situation of the HP sold in Brazil, some points must be considered to reach the full potential and benefits from it:

(1) Develop Pharmacopoeia Monographs for Brazilian native plants including DNA barcode. In the last years, DNA barcoding from different organisms has gained rapid acceptance in the scientific community from various fields, including studies of plant identification (CBOL Plant Working Group, 2009). Despite DNA barcoding being relatively new, pharmacopoeias around the world, such as Ayurveda (Indian), British, Chinese, and Korean, have already introduced protocols for DNA barcoding authentication. In the last decade, significant advances regarding DNA sequencing were made, making this technique more viable to be used for the purpose of species identification in complex samples. Herbal products may contain several species in their composition, and even when the product is declared to contain only one species, we may encounter adulteration by the addition of other species. The methodological advances in high-throughput sequencing (HTS) were crucial to the analysis of those samples. Different from Sanger sequencing, which has the limitation of only analyzing one sample per reaction, HTS made the parallel sequencing of thousands of samples possible simultaneously in a cost-effective manner, besides being more sensitive and faster (Coghlan et al., 2012; Ivanova et al., 2016). Combining the use of DNA barcoding and HTS, the metabarcoding technique was developed. Metabarcoding is the use of universal PCR primers to mass-amplify barcodes from DNA extracted from complex samples (de Boer et al., 2015). This technique has been used more and more, allowing the study of thousands of samples of HPs simultaneously to evaluate their authenticity and safety (Ivanova et al., 2016; Seethapathy et al., 2019; Urumarudappa et al., 2020). Despite its benefits, DNA barcode is not able to identify which part of the plant was used for the preparation of HPs or even if the material was well preserved. Therefore, DNA barcoding should be used as a complementary method to improve the quality and safety of HPs (de Boer et al., 2015; Palhares et al., 2015).
(2) List the resilient useful Brazilian species and create a database with different information about them. Adulteration and substitutions may be occurring for several reasons, for example, cross-contamination and fraudulent practices for financial gain. The problem might also be amplified by a lack of inspection by health agencies. Another cause for the substitution of a native species, or even the plant parts used for the HP preparation, is the lack of knowledge about the plants, caused by the genetic and cultural erosion occurring in some regions in Brazil. In fact, studies performed by us in recently deforested regions of Minas Gerais have shown that the population no longer knows or uses native species from traditional medicine (Brandão and Montemór 2008; Prates et al., 2020). In 2010, we created the Dataplamt database (www.dataplamt.org.br) with information about traditional uses of Brazilian plants, recovered from references published until 1950. After this date, the use of native plants has declined, as a consequence of the installation of foreign pharmaceutical industries in Brazil (Manhã et al., 2008). To date, Dataplamt has information on 3,400 Brazilian plant species with more than 150 different uses. In another recent work, we have developed a strategy to identify resilient useful species, that is, plants used for the same purpose along the five centuries of the written history of Brazil (Ricardo et al., 2018). We argue that a database with traditional, pharmacopoeia, chemical, pharmacological, toxicological, and DNA barcode (Liu et al., 2017; Wong et al., 2018) data for these resilient species should be created, in order to support actions on pharmacovigilance. An international cooperative network for researches studying these plants, specially those that are commercialized in other countries, could contribute to filling the lacks.
(3) It is imperative to respect the regulations to protect Amerindian and other traditional communities. Brazil has specific legislation that protects traditional knowledge but there have been few institutional advances to achieve these objectives (Hanazaki et al., 2018). In August 2020, Brazil ratified the Nagoya Protocol, a multilateral agreement accessory to the Convention on Biological Diversity, created during the United Nations Conference on Environment and Development held in Rio de Janeiro in 1992 (Eco-92) (Agência Brasil, 2020). This international legislation regulates access and benefit-sharing and aims at the intellectual property protection of genetic resources and traditional knowledge. To defined measures to comply with the rules and especially to protect Amerindian knowledge are urgent and necessary.
(4) It is necessary to teach the society to value and protect the Brazilian biodiversity. Ichim (2019) showed that adulteration in HP marketed in Asia and Africa is less common than in other countries. This occurs because in these continents traditional medicines are strongly recognized by population. Since 2014, our research group is also doing a set of works with school teachers and students living in small cities, showing them the importance of plant biodiversity and traditional knowledge associated with them (Prates et al., 2020). It is strongly necessary to promote such activities in all regions of Brazil; nobody protects or values what they do not know!

## Conclusion

The adulteration found in Brazilian HP reflects a trend that can be seen throughout the world. The points listed can contribute to improving their quality, especially those from native Brazilian species.

## Author Contributions

All authors contributed to the bibliographic survey and preparation of the manuscript, each writing about their area of knowledge. MGLB was also responsible for coordinating the group and funding acquisition.

## Funding

The study was funded by Fundação de Apoio a Pesquisa do Estado de Minas Gerais (FAPEMIG/PPM00691-16) and Conselho Nacional de Desenvolvimento Científico e Tecnológico (CNPq/310389/2017-8).

## Conflict of Interest

The authors declare that the research was conducted in the absence of any commercial or financial relationships that could be construed as a potential conflict of interest.
